# Oncological considerations of skin-sparing mastectomy

**DOI:** 10.1186/1477-7800-3-14

**Published:** 2006-05-25

**Authors:** GH Cunnick, K Mokbel

**Affiliations:** 1 Wycombe General Hospital, Queen Alexandra Road, High Wycombe, Buckinghamshire, HP11 2TT, UK; 2 St. George's Hospital, Blackshaw Road, Tooting, London, SW17 0QT, UK

## Abstract

**Aim:**

To review evidence concerning the oncological safety of performing skin-sparing mastectomy (SSM) for invasive breast cancer and ductal carcinoma in situ (DCIS). Furthermore, the evidence concerning RT in relation to SSM and the possibility of nipple preservation was considered.

**Methods:**

Literature review facilitated by Medline and PubMed databases.

**Findings:**

Despite the lack of randomised controlled trials, SSM has become an accepted procedure in women undergoing mastectomy and immediate reconstruction for early breast cancer. Compared to non-skin-sparing mastectomy (NSSM), SSM seems to be oncologically safe in patients undergoing mastectomy for invasive tumours smaller than 5 cm, multicentric tumours, DCIS or risk-reduction. However, the technique should be avoided in patients with inflammatory breast cancer or in those with extensive tumour involvement of the skin in view of the high risk of local recurrence. SSM with nipple areola complex (NAC) preservation appears to be oncologically safe, provided the tumour is not close to the nipple and a frozen section protocol for the retro-areolar tissue is followed. Although radiotherapy (RT) does not represent a contraindication to SSM, the latter should be used with caution if postoperative RT is likely, since it detracts from the final cosmetic outcome.

## Background

**Figure 1:**
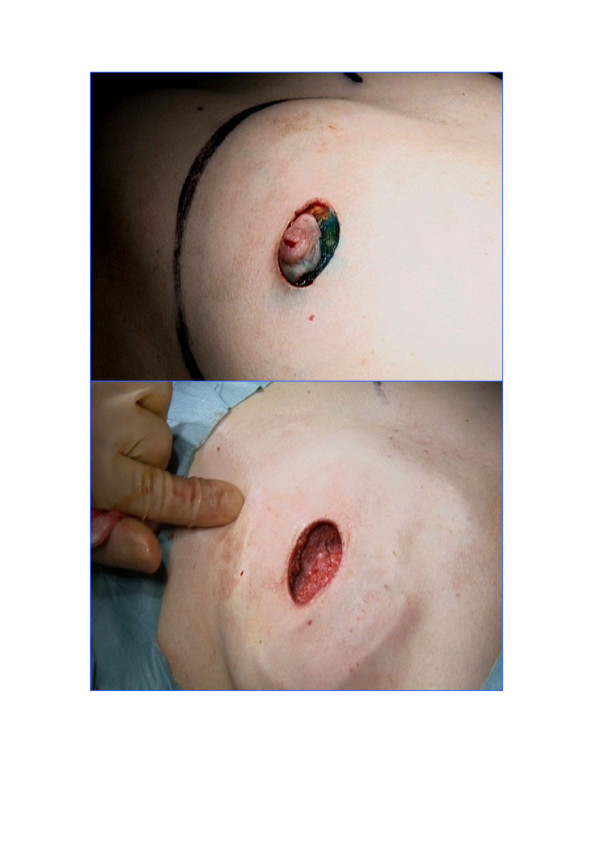
The peri-areola incision of standard skin-sparing mastectomy.

**Figure 2:**
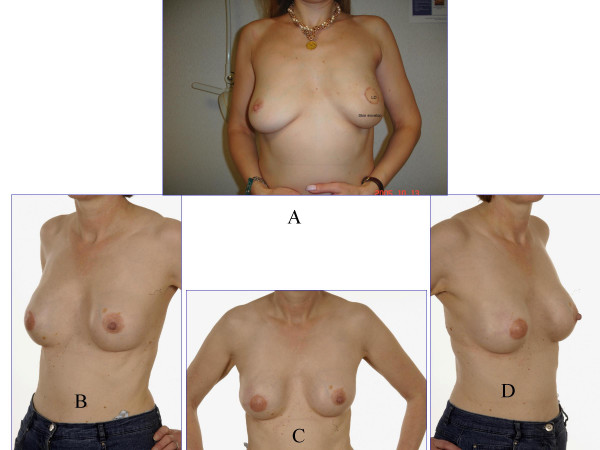
A: This 40 year old woman had left SSM and immediate LD flap reconstruction for DCIS last year. She is due to have nipple reconstruction shortly. B, C, and D: This 52 year old woman had right SSM and LD flap reconstruction followed by nipple reconstruction using a local skin flap (Trefoil technique) and subsequent tattooing 3 years ago.

**Figure 3:**
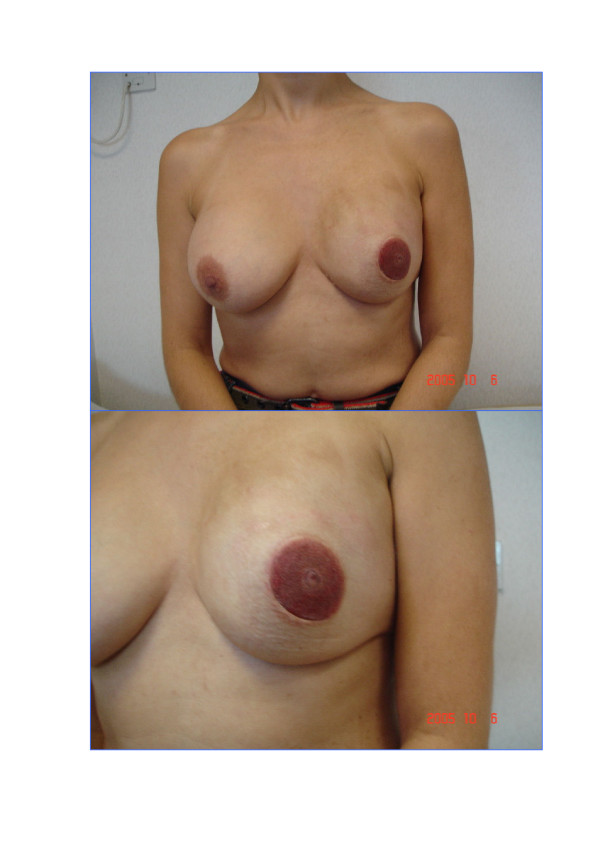
This 42 year old doctor had left skin-sparing mastectomy and immediate breast reconstruction using the latissimus dorsi myocutaneous flap and implant 2 years ago, followed by a nipple reconstruction using the nipple-sharing technique and tattooing.

Immediate or delayed breast reconstruction following conventional non-skin sparing mastectomy (NSSM) often results in prominent scars on the new breast and a paddle of skin that is of a different colour and texture. Skin-sparing mastectomy (SSM) preserves most of the overlying skin (figures 1 and 2) during an immediate breast reconstruction (IBR) thus leading to a superior aesthetic outcome (figures 2 and 3). It also reduces the need for contralateral breast adjustment in order to achieve symmetry [1]. In a recent survey in the UK, 35 out of 95 breast surgeons avoided SSM because of concerns over oncological safety or uncertainty of the benefits or indications [2]. In view of these uncertainties, this article reviews the controversies surrounding SSM, using Medline and PubMed databases and the keywords 'skin-sparing mastectomy', 'subcutaneous mastectomy' and 'immediate breast reconstruction'.

## Oncological concerns

The main oncological concern in both SSM and NSSM relates to the possibility of leaving residual tumour within the skin envelope which may manifest later as local recurrence (LR). Indeed, Ho et al [3] performed histological examinations of the skin and subcutaneous tissue of 30 NSSM specimens and found that the skin flaps (excluding the NAC) were involved in 23% (7 of 30) of cases. In 5 cases, the skin involved was situated directly over the tumour. Unfortunately, many of the studies addressing the issue of LR following SSM have not followed-up patients long enough for it to be seen. In addition, there is a significant lack of prospective data and nearly all studies are from single institutions. However, the incidence of LR following SSM for invasive breast cancer has been retrospectively investigated by several authors and is summarised in table 1[4-15]. The largest series [5] observed 539 patients over a period of 65 months. 30.6% of cases had non-invasive disease. The LR rate was 5.5%. Furthermore, LR was related to tumour size, grade, nodal status and lymphovascular invasion. LR rates were similar for T2 and T3 tumours, although this does not correlate with the laboratory observations of Ho et al [3]. Other studies have found even lower LR rates. A study of 176 breast cancers treated by SSM and IBR reported a LR rate of 4.5% after a median follow-up of 73 months [6]. After 9.8 years' follow-up of 177 SSMs, Spiegel and Butler [7] reported a LR rate of 5.6%. In a series of only T1 and T2 breast cancers, a LR of 6.2% for 372 SSMs (23/372) was reported after only 26 months' follow-up [8]. Similarly, Kroll observed a 7.0% LR rate (8/114 cases) in patients with T1/T2 tumours treated by this method [9]. In addition, they found a similar LR rate of 7.5% (3/40) in women treated by NSSM and IBR. One study found no LRs in a series of 50 patients after a follow up of 51 months, although there were 5 distant recurrences [4]. Many of these studies found that tumour size, stage, lymph node positivity and poor differentiation were all risk factors for LR. Unfortunately, the frequency of giving radiotherapy (RT) and chemotherapy was not uniform across these series which may explain the variable LR rates.

**Table 1:** Oncological safety of skin-sparing mastectomy for invasive breast cancer – summary of recent studies.

**Authors**	**Year**	**Sample size**	**L.R. (%)**	**F/U (months)**	**Notes**
Slavin et al^10^	1998	51	2.0	45	26 DCIS cases.
Newman et al^8^	1998	372	6.2	26	T1/T2 tumours.
Simmons et al^13^	1999	77	3.9	60	
Toth et al^4^	1999	50	0	51.5	
Kroll et al^9^	1999	114	7.0	72	T1/T2 tumours.
Rivadeneira et al^12^	2000	71	5.1	49	
Foster et al^11^	2002	25	4.0	49	Locally advanced.
Medina-Franco et al^6^	2002	176	4.5	73	
Spiegel and Butler^7^	2003	177	5.6	118	
Carlson et al^5^	2003	539	5.5	65	30.6% DCIS.
Gerber et al^14^	2003	112	5.4	59	
Downes et al^15^	2005	38	2.6	53	'High risk tumours'

Abbreviations: L.R. = local recurrence; F/U = follow-up interval.

In a large series of standard mastectomies and wide local excisions by Fisher, the LR rate of breast cancer after NSSM in tumours up to 4 cm was found to be 10% after 20 years follow-up [16]. This approximately equates to 0.5% per year. The studies outlined above have demonstrated that the average LR rate following SSM and IBR is similar to that reported for NSSM, although the period of follow-up duration for SSM is shorter.

Unfortunately, it is unlikely that large multi-centre randomised studies comparing NSSM and SSM will take place. However, although the above studies are retrospective and relatively small, there is reasonable evidence that SSM is a safe oncological operation for T1, T2 and multicentric tumours. Moreover, there is evidence that SSM combined with IBR does not significantly delay adjuvant therapy, as some clinicians had feared [17]. Many T1/T2 tumours, however, can be treated adequately by breast conservation surgery followed by RT [16,18] which may be preferred to IBR if cosmesis is a high priority. Many patients requiring mastectomy have T3 tumours, yet the evidence for the safety of SSM for these tumours is less clear, but encouraging. In a study of 38 patients with tumours considered to be at high risk of LR, only one case (2.6%) developed a LR after SSM and IBR after 53 months of follow-up, despite 10 (26%) systemic recurrences [15]. Yet a further option for patients with T3 tumours is to administer neoadjuvant chemotherapy in an attempt to shrink the tumour. If shrinkage does occur, it may facilitate performance of SSM in a breast which may otherwise have required a NSSM. Alternatively, it may even facilitate breast conservation surgery, avoiding the need for a mastectomy altogether.

## Surgical considerations

Although the incision(s) differ, the technique of dissecting the skin flaps during SSM is similar to that of NSSM. Native skin flap necrosis (partial or complete) has been estimated to occur in 11% of cases and is similar in SSM and NSSM [19]. Since the main difference between SSM and NSSM is that the standard incision for SSM is circumareolar rather than a large ellipse [20,21], the only breast skin excised is the nipple areola complex (NAC) (see figure 1 and 2). SSM combined with sentinel node biopsy (SNB) and/or axillary node clearance (ANC) can be safely performed through this incision [1,21], although some prefer to perform the axillary procedure through a separate small incision in the axilla [22]. The latter is hidden from view, maintaining cosmesis. Some surgeons perform an intra-operative histopathological assessment of the sentinel node (frozen section or imprint cytology) and perform an immediate ANC if the SNB is positive for malignancy. An alternative approach is to perform a day-case sentinel node biopsy one or two weeks before the mastectomy so that nodal status is known. Additionally, to maximise a good cosmetic outcome, the dissection of the lower skin flap should not continue beyond the inframammary fold, so that the final shape of the reconstruction will be very similar to the original breast [23]. In patients with large ptotic breasts, SSM can be performed through the standard incisions used for reduction mammoplasty [24]. Symmetry may be achieved by performing a simultaneous, or delayed, contralateral reduction mammoplasty using similar incisions.

## SSM for DCIS

A standard mastectomy (NSSM) for DCIS achieves cure rates of approximately 98% (LR rate of 1.4% and breast cancer-specific mortality of 0.59%) [25]. Furthermore, RT is not usually required afterwards. Consequently, SSM and IBR would appear to be an ideal choice for women undergoing mastectomy and reconstruction for DCIS, since postoperative RT side-effects are no longer a concern. In one series [26], 93 out of 95 patients (98%) who had undergone SSM and IBR for DCIS were alive and disease-free after 3.7 years of follow-up. In 35 cases, the margins were closely examined by performing intra-operative specimen radiography and histological examination of serial sections. Margins were found to be negative in 77% of the cases. In the remainder, further tissue was removed. None of these 35 cases developed LR. 3 of the 58 other cases developed LR. The overall LR rate was, therefore, 3 out of 93 (3%). A larger series by Carlson et al. included 175 cases of DCIS5 in which there was only one LR (0.6%) after 65 month' follow-up. None of 26 DCIS patients developed LR in the study by Slavin et al. after 45 months [10]. Finally, in a longer-term follow-up retrospective study of 44 patients who underwent SSM and IBR for DCIS, there were no local or distant recurrences by 9.8 years [7]. These retrospective studies have all demonstrated that SSM and IBR for DCIS is oncologically safe with low recurrence rates (see table 2). However, prospective data to confirm these findings is not available.

**Table 2:** Outcome of skin sparing mastectomy for DCIS – summary of recent studies.

**Authors**	**Year**	**Sample size**	**L.R. (%)**	**Follow-up**
Slavin et al^10^	1998	26	0	45 months
Rubio et al^26^	2000	95	3	3.7 years
Spiegel & Butler^7^	2003	44	0	9.8 years
Carlson et al^5^	2003	175	0.6	65 months

Abbreviations: L.R. = local recurrence

## The oncological safety of nipple areola complex preservation

Although IBR following SSM may offer cosmetic benefits over NSSM, removal of the nipple areola complex (NAC) significantly impacts on the final outcome. Patients are offered a subsequent delayed nipple reconstruction and areola tattooing or a prosthetic NAC. The NAC is removed because of the belief that the NAC and its adjacent ducts may contain tumour cells which have spread distally along the ducts from the primary tumour. This concept was based on older studies that had demonstrated occult tumour in the region of the NAC [23]. Recent evidence has shown that the risk of tumour involvement of the NAC has been overestimated [27-29]. This has led some surgeons to attempt preservation of the NAC in view of the cosmetic benefit. In a retrospective series of 286 SSM specimens, 16 (5.6%) were found to contain tumour in the NAC [27]. Nodal positivity, subareolar tumour location and multicentricity were significant risk factors for NAC involvement. If multicentric and subareolar tumours were excluded, the NAC was only involved in 3% of cases. Another series of 140 mastectomies also found tumour size and nodal positivity to be risk factors for NAC involvement [28]. Furthermore, the primary tumour was situated within 2.5 cm of the areola in all 22 cases in which the NAC was positive. A retrospective study involving 217 mastectomy specimens by Simmons et al. reported NAC tumour involvement in 23 cases (10.6%) [29]. It was also found that only 6.7% of small tumours with up to two positive lymph nodes only had NAC involvement. In contrast to these modest figures, one report found that the NAC was involved in up to 58% of mastectomy specimens [30]. Reasons for this difference are unclear, although this is the only study to publish such a high probability of NAC involvement. More recently, Gerber et al. performed 112 SSMs in women whose breast cancer was more than 2 cm from the NAC [14]. Histological examination of intra-operative frozen sections of the subareolar tissue was performed in an attempt to predict NAC involvement. The biopsies were negative for tumour in 61 (54.5%) cases, thus enabling NAC preservation. The NAC was excised in the other 51 cases. The cosmetic results after SSM and IBR (using LD or TRAM flaps) were independently evaluated as excellent or good in 91% (102/112) of patients and were significantly better after preservation of the NAC (P = 0.001). Six (5.4%) recurrences occurred in 112 patients who underwent SSM compared with 11 (8.2%) of 134 patients who had undergone NSSM during the same 6-year period. Only one LR occurred in the NAC preservation group. Therefore, it would appear oncologically safe to perform SSM with NAC preservation, provided the tumour is not close to the nipple and a frozen section protocol is followed. Unfortunately, up to one-third of patients lose part or all of the NAC after this operation due to impaired NAC blood supply [31], although Crowe et al [32] found this complication to be relatively uncommon. Differences are likely to be due to surgical technique. In the latter study, 54 SSMs in 44 patients were performed in which the NAC was clinically thought to be disease-free. Nipple core biopsy frozen sections were performed. 6 out of 54 biopsies were positive, necessitating conversion to conventional SSM. Of the remaining 48 NAC-sparing SSMs, 45 had no skin loss and only the remaining 3 cases had partial NAC loss. In a different series of 54 SSMs of which 34 had NAC preservation [33], the skin loss was higher in the latter group although LR was similar (8.3% when NAC excised; 7.1% when NAC preserved).

An alternative to NAC-sparing SSM is to remove the nipple but preserve the areola – a technique called areola-sparing mastectomy (ASM). The concept of this technique is supported by the findings of Simmons et al. who found that the areola itself was only involved in 2 of the 23 cases of positive NACs [29]. This represented 0.9% of all the mastectomy specimens. Access for ASM is facilitated by medial and lateral extensions to the incision encircling the nipple. This may achieve a superior cosmetic outcome compared to conventional SSM, only requiring a subsequent nipple reconstruction, if requested by the patient. Unfortunately, nipple reconstruction using one of the conventional local flap technique is problematic in this situation. The only published series of areola-sparing SSM was also by Simmons [34] who reported 17 cases with only a single complication (wound infection) over a 20-month period.

## RT after SSM

Most women undergoing mastectomy for breast cancer do not require post-operative RT. However, patients with several positive regional lymph nodes and/or large tumours are offered RT in view of the proven reduction in loco-regional recurrence and improved survival [35]. Similarly, RT is also indicated in some patients who have undergone SSM and IBR. Recently, the incidence of post-mastectomy RT has been increasing [36]. Post-reconstruction RT is unfortunately associated with local complications, thus causing some debate as to the safety of performing SSM and IBR in women who are likely to require this treatment. There is a lack of prospective trials concerning the use of RT with SSM and most of the published evidence is derived by enthusiasts from single-centres.

Although results from individual series vary considerably, it appears that the complications of RT following immediate breast reconstruction occur in a high proportion of patients [37]. A study of immediate TRAM reconstructions showed the commonest complications were fat necrosis (16%) and radiation fibrosis (11%) [38], although this population underwent autologous reconstructions. Fat necrosis leads to volume loss and hardening of the reconstructed breast and particularly occurs when RT is given after IBR using free tissue transfer of skin and fat only (e.g. deep inferior epi-gastric perforator; DIEP flap). The main concern regarding RT in the reconstructed breast, however, is related to the use of implants, either alone or in conjunction with a flap reconstruction. The fibrosis in particular often causes subsequent shrinkage of the reconstructed breast around the implant – termed 'capsular contraction'. One study compared 39 irradiated implant reconstructed breasts with 338 non-irradiated reconstructions and found a significant negative effect on the reconstructive outcome with implants [39], the main complications being capsular contracture and post-operative pain. 43% of patients underwent a subsequent capsulotomy. Capsular contraction results in poor aesthetic outcomes in many cases, sometimes even requiring further flap surgery. This has led some surgeons to recommend that IBR using implants, including SSM, be avoided if it is known that a patient is likely to require postoperative RT [37]. Alternative approaches are to either use an autologous IBR, to deliberately oversize the reconstruction, or to place a temporary tissue expander under the SSM skin envelope deep to the pectoralis major muscle – following RT, the delayed reconstruction can be performed using a myocutaneous flap and implant after removing the tissue expander [23]. Other surgeons do not hold this view. In another retrospective study [40], 68 IBRs who received postoperative RT were compared with 75 IBRs who did not. It was found that although capsular contracture rates (68% v 40%, respectively), cosmetic outcome (good or better in 80% v 88%) and patient satisfaction (67% v 88%) all favoured the group who avoided RT, 72% of those irradiated said that they would still chose the same form of reconstruction again.

Hultman and Daiza investigated the effects of previous RT on subsequent SSM and IBR in 37 breasts, although not all patients had received previous RT [41]. TRAM and LD flaps and implant reconstructions were all included. 9 patients (24%) had a SSM flap complication of which 5 required re-operations. Previous irradiation and diabetes were found to be significant risk factors for complications. The issue of pre-operative RT and SSM was also investigated by Disa et al [42]. Their study included only 11 patients who underwent SSM and IBR after developing LRs following previous breast conservation surgery and RT. They observed that all the flaps survived, one patient developed partial thickness skin flap loss and two developed capsular contractures, leading them to conclude that SSM and IBR can be safely performed in previously irradiated breasts. Furthermore, Benediktsson and Perbeck have shown that RT does not significantly compromise the skin circulation of the breast [43]. Therefore, as long as a slightly higher complication rate is accepted and the patients are fully informed, it would appear safe for women to undergo SSM and IBR after previous breast RT. However, these studies are small and larger studies with longer follow-up are required to verify these findings.

## Abbreviations

skin-sparing mastectomy (SSM); ductal carcinoma in situ (DCIS); immediate breast reconstruction (IBR); non-skin-sparing mastectomy (NSSM); nipple areola complex (NAC); areola-sparing mastectomy (ASM); radiotherapy (RT); local recurrence (LR)
